# Statins are underused in recent-onset Parkinson's disease with increased vascular risk: findings from the UK Tracking Parkinson's and Oxford Parkinson's Disease Centre (OPDC) discovery cohorts

**DOI:** 10.1136/jnnp-2016-313642

**Published:** 2016-11

**Authors:** Diane M A Swallow, Michael A Lawton, Katherine A Grosset, Naveed Malek, Johannes Klein, Fahd Baig, Claudio Ruffmann, Nin P Bajaj, Roger A Barker, Yoav Ben-Shlomo, David J Burn, Thomas Foltynie, Huw R Morris, Nigel Williams, Nicholas W Wood, Michele T M Hu, Donald G Grosset

**Affiliations:** 1 Department of Neurology, Institute of Neurological Sciences, Glasgow, UK; 2 School of Social and Community Medicine, University of Bristol, Bristol, UK; 3 Oxford Parkinson's Disease Centre, Nuffield Department of Clinical Neurosciences, University of Oxford, Oxford, UK; 4 Department of Neurology, Queen's Medical Centre, Nottingham, UK; 5 Clinical Neurosciences, John van Geest Centre for Brain Repair, Cambridge, UK; 6 Institute of Neuroscience, University of Newcastle, Newcastle upon Tyne, UK; 7 Sobell Department of Motor Neuroscience, UCL Institute of Neurology, London, UK; 8 Department of Clinical Neuroscience, UCL Institute of Neurology, London, UK; 9 Psychological Medicine and Clinical Neurosciences, MRC Centre for Neuropsychiatric Genetics and Genomics, Cardiff University, Cardiff, UK; 10 Department of Molecular Neuroscience, UCL Institute of Neurology, London, UK; 11 UK Clinical Consortium, 72 Clinical Centres across the UK

## Abstract

**Background:**

Cardiovascular disease (CVD) influences phenotypic variation in Parkinson's disease (PD), and is usually an indication for statin therapy. It is less clear whether cardiovascular risk factors influence PD phenotype, and if statins are prescribed appropriately.

**Objectives:**

To quantify vascular risk and statin use in recent-onset PD, and examine the relationship between vascular risk, PD severity and phenotype.

**Methods:**

Cardiovascular risk was quantified using the QRISK2 calculator (high ≥20%, medium ≥10 and <20%, low risk <10%). Motor severity and phenotype were assessed using the Movement Disorder Society Unified PD Rating Scale (UPDRS) and cognition by the Montreal cognitive assessment.

**Results:**

In 2909 individuals with recent-onset PD, the mean age was 67.5 years (SD 9.3), 63.5% were men and the mean disease duration was 1.3 years (SD 0.9). 33.8% of cases had high vascular risk, 28.7% medium risk, and 22.3% low risk, while 15.2% of cases had established CVD. Increasing vascular risk and CVD were associated with older age (p<0.001), worse motor score (p<0.001), more cognitive impairment (p<0.001) and worse motor phenotype (p=0.021). Statins were prescribed in 37.2% with high vascular risk, 15.1% with medium vascular risk and 6.5% with low vascular risk, which compared with statin usage in 75.3% of those with CVD.

**Conclusions:**

Over 60% of recent-onset PD patients have high or medium cardiovascular risk (meriting statin usage), which is associated with a worse motor and cognitive phenotype. Statins are underused in these patients, compared with those with vascular disease, which is a missed opportunity for preventive treatment.

**Trial registration number:**

GN11NE062, NCT02881099.

## Background

Parkinson's disease (PD) and cardiovascular disease (CVD) become more prevalent with advancing age. CVD is therefore likely to affect a large number of individuals with PD. A variety of clinical, imaging and pathological studies in elderly individuals without PD,^1^ ^2^ as well as smaller PD studies,^3–10^ show links between established vascular disease and vascular risk factors, and gait and cognitive impairment. A combination of Lewy body and vascular pathology may create a mixed clinical phenotype, and explain some of the variation in the responsiveness of the motor and cognitive features to antiparkinsonian therapy.

Vascular preventive treatment is well established. Primary prevention is recommended when an individual's calculated 10-year vascular risk is 10% or more^11^ and involves the use of cholesterol-lowering therapy (mainly with HMG-CoA reductase inhibitors, commonly referred to as statins) and management of other vascular risk factors such as hypertension. Secondary prevention (after a vascular event) similarly involves the use of statins, as well as antihypertensive and antiplatelet therapy. Additionally, statins are of particular interest in PD as possible neuroprotectants, given their beneficial role in the attenuation of inflammatory responses, including the production of tumour necrosis factor α, nitric oxide and superoxide; the reduction in the accumulation of α-synuclein; and alteration of dopamine D1/D2 receptor modulation.^12^ However, varying rates of statin usage are reported in patients with vascular risk and vascular disease,^13–18^ and it is not known whether PD patients have equitable access to statins.

We therefore studied cardiovascular risk and CVD rates in recent-onset PD, in relation to clinical phenotype, more specifically the motor and cognitive features, and the use of statins in these patients.

## Methods

### Participants

Study participants were enrolled prospectively in either the UK Tracking Parkinson's study, or the Oxford Discovery study. Participants were recruited from February 2012 to May 2014 in the Tracking Parkinson's study and from September 2010 to October 2015 in the Oxford Discovery study. Our analysis is based on the baseline data from these large multicentre studies, whose protocols including inclusion/exclusion criteria are detailed elsewhere.^19^ ^20^ In brief, in both studies, cases with a clinical diagnosis of PD were recruited, fulfilling Queen Square Brain Bank criteria, with written consent, approval of multicentre regional ethics committees and in compliance with national legislation and the Declaration of Helsinki. For the current analysis, cases with normal functional dopaminergic imaging performed after study entry, and cases with a revised diagnosis at their latest follow-up visit, were excluded. When assessing the clinical correlates of CVD, we further excluded cases with any features that were possibly atypical or unusual at baseline assessment, including an unusual presentation, symptom, sign, progression or response to medication, to reduce any effect from a possible alternative diagnosis than PD (eg, vascular parkinsonism).

### Measurement instruments

Established vascular diagnoses and risk factors were collected from self-report completed at clinic attendance, and was performed at the same time as, and therefore with input from, physician/nurse access to medical records. The individual vascular factors collected are the standard risk factor variables recommended by the National Institute of Clinical Excellence (NICE) in the UK to clinically assess vascular risk.^21^ The 10-year future cardiovascular risk was then calculated using the QRISK2-2015 prediction algorithm,^22^ also recommended by NICE,^11^ which computes risk based on demographic and comorbid vascular features for example, age, smoking status, ethnicity, systolic blood pressure, body mass index, treated hypertension and type 2 diabetes. The calculation is only appropriate in patients aged under 85 years and without a previous vascular event. Since national treatment thresholds were reduced recently from 20% to 10% calculated risk,^11^ statin implementation was examined against both of these thresholds, and designated as high (≥20%), medium (≥10 and <20%) or low (<10%) risk. Vascular preventive medications, including lipid-lowering, antiplatelet, anticoagulant and antihypertensive therapies, were identified from medication histories, using British National Formulary classifications.

Motor function was scored according to Part 3 of the Movement Disorder Society Unified PD Rating Scale (UPDRS 3), and was used to define motor subtype using a predetermined formula which uses these variables to define tremor dominant (TD) and postural instability gait difficulty (PIGD) phenotypes.^23^ Cognition was assessed by the Montreal cognitive assessment (MoCA), adjusted for education years according to standard methods (1 point added to the total score if education years were ≤12, to a maximum score of 30) and categorised as normal cognition (24–30), mild cognitive impairment (22–23) or dementia (<22).^24^ Levodopa equivalent daily dose (LEDD) was calculated using an established formula.^25^

### Statistical analysis

Generalised linear modelling was used to assess clinical variables across vascular risk categories, adjusting for multiple covariates (age, sex, disease duration and coffee use), with heterogeneity and trend p values calculated when there were more than two categories. The linearity of the continuous confounders (age and disease duration) was tested using fractional polynomials in univariate models and then transformed if they showed evidence of non-linearity. Regression models used were: multinomial logistic for motor subtype analysis (using TD as the baseline); ordered logistic for categorised MoCA and smoking status; linear for age, disease duration, MoCA total, LEDD and UPDRS 3; and logistic for sex. The main analysis excluded those with a revised diagnosis, those diagnosed >3.5 years ago and those without available QRISK2 prediction algorithm or medication data. An additional sensitivity analysis was performed imputing missing outcome and exposure data. In this analysis, MoCA, motor phenotype and UPDRS 3 scores were calculated using expected scores where at least 80% of the questions in each scale were answered. Any remaining missing data were imputed using the chained equation approach to multiple imputation, creating 10 imputed data sets. Adjusted p values of <0.05 were considered significant. IBM SPSS Statistics for Windows, V.22.0, Armonk, New York, USA, and STATA V.13 were used.

## Results

**Figure 1:**
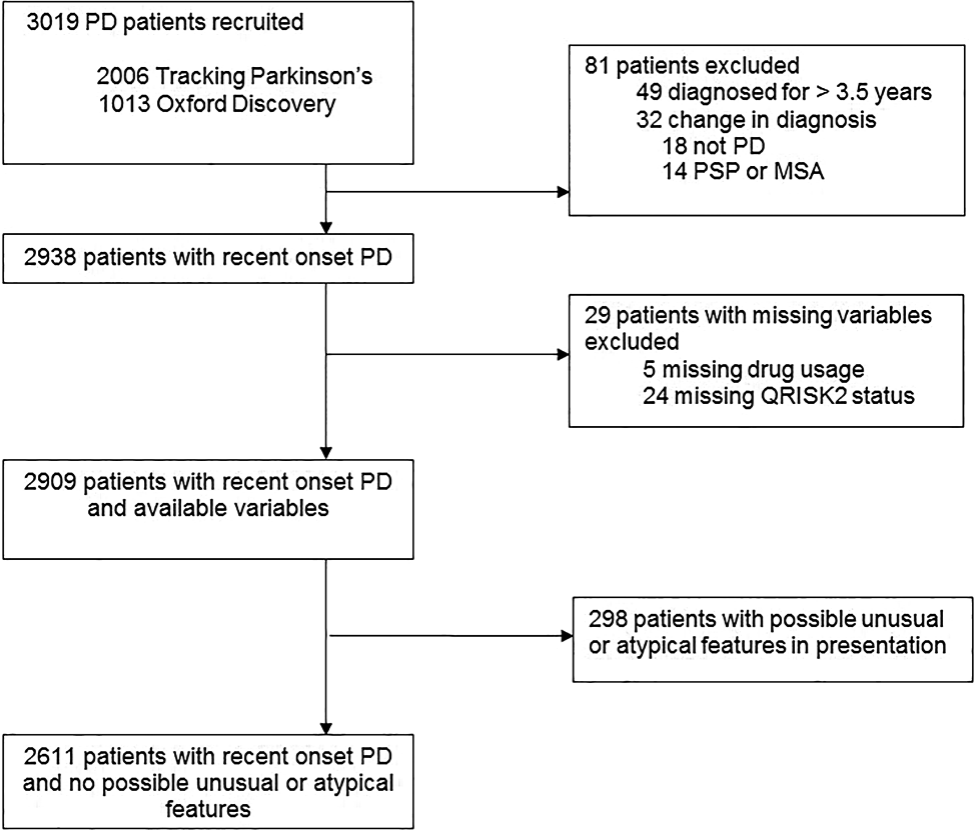
Disposition of cases recruited to the study, and reasons for exclusion. The main analysis was performed in 2909 cases; clinical correlates were examined in 2611 cases, to reduce any effect of possible diagnostic inaccuracy. PD, Parkinson's disease, PSP, progressive supra nuclear palsy, MSA, multiple system atrophy.

Out of 3019 cases (Tracking Parkinson's 2006, Oxford Discovery 1013), 110 (3.6%) were excluded for the following reasons: revised diagnosis (n=32), disease duration >3.5 years (n=49), missing statin usage (n=5) and missing QRISK2 status (n=24; figure 1). In the remaining 2909 cases, the mean age was 67.5 years (SD 9.3), mean disease duration 1.3 years (SD 0.9) and 65.3% were men. Further baseline characteristics are summarised in table 1.

**Table 1:** Demographic and clinical characteristics of 2909 recent-onset Parkinson's disease cases

Variable	Result
Age	67.5 (9.3)
Male sex	1898 (65.3%)
Disease duration (years)	1.3 (0.9)
UPDRS 3	24.2 (12.1)
Motor subtype	
TD	1303 (48.1%)
PIGD	1061 (39.2%)
Indeterminate	344 (12.7%)
MoCA	
Total score	25.1 (3.5)
Normal	1972 (72.1%)
MCI	370 (13.5%)
Dementia	394 (14.4%)
Antiparkinsonian medication	
Drug naïve	314 (10.8%)
Levodopa	1776 (61.2%)
Dopamine agonist	873 (30.0%)
MAOB inhibitor	723 (24.9%)
COMT inhibitor	67 (2.3%)
Anticholinergic	38 (1.3%)
Amantadine	28 (1.0%)
LEDD	292 (214)

Data are mean (SD) or number (percentage).

COMT, catechol-*O*-methyl transferase; LEDD, levodopa equivalent daily dose; MAOB, monoamine oxidase type B; MCI, mild cognitive impairment; MoCA, Montreal cognitive assessment; PIGD, postural instability gait difficulty; TD, tremor dominant; UPDRS 3, Movement Disorder Society unified Parkinson's disease rating scale part 3.

Established vascular disease was present in 15.2% of cases (table 2). Hypertension was present in 34.4%, high cholesterol in 32.4%, diabetes in 8.6% and current cigarette smoking in 3.2%. Antihypertensive medication was the most commonly prescribed preventive cardiovascular therapy at 39.5%, while lipid-lowering medication was prescribed in 30.4%. Statins were the most common lipid-lowering medication (97.9% of cases on lipid-lowering therapy). Other cardiovascular risk factors and preventive medications are listed in table 2.

**Table 2:** Vascular disease, risk factors and treatment in 2909 recent-onset Parkinson's disease cases

Variable	Result
Vascular diagnosis	441 (15.2%)
Angina	269 (9.3%)
Myocardial infarction	134 (4.6%)
Transient ischaemic attack/stroke	150 (5.2%)
Vascular risk factors	
Diabetes	249 (8.6%)
Type 1	13 (0.5%)
Type 2	236 (8.2%)
High cholesterol	936 (32.4%)
Hypertension	992 (34.4%)
Rheumatoid arthritis	69 (2.4%)
Body mass index (kg/m^2^)	27.1 (4.7)
Blood pressure, systolic/diastolic	140 (20)/80 (11)
Orthostatic hypotension	525 (18.5%)
Smoking	
Non-smoker	1539 (57.9%)
Ex-smoker	1023 (38.5%)
Current smoker	96 (3.2%)
Light	48 (1.8%)
Moderate	33 (1.3%)
Heavy	15 (0.6%)
Current coffee intake (cups per day)	
Less than 1	257 (9.6%)
1	194 (7.2%)
2–3	719 (26.7%)
4 or more	1522 (56.5%)
Cardiovascular medication	
Lipid lowering	885 (30.4%)
Statins	866 (97.9%)*
Other	19 (2.1%)*
Antihypertensive	1150 (39.5%)
Antiplatelet	430 (14.8%)
Anticoagulant	122 (4.2%)

*Percentage of total lipid lowering.

Data are mean (SD) or number (percentage).

The QRISK2 score (ie, 10-year future cardiovascular risk) was low in 648 cases (22.3%), medium in 836 cases (28.7%) and high in 984 cases (33.8%; table 3). Statins were prescribed in a minority of those with a vascular risk indication for their use (15.1% of those with medium vascular risk, and 37.2% of cases with high vascular risk). In contrast, statins were prescribed in 75.3% of cases with established vascular disease.

**Table 3:** Vascular preventive medication usage according to indication in 2909 recent-onset Parkinson's disease cases

Medication	Primary prevention			Secondary prevention
	QRISK2 <10% =648 (22.3%)	QRISK2 ≥10% and <20% n=836 (28.7%)	QRISK2 ≥20% n=984 (33.8%)	n=441(15.2%)
Statin	42 (6.5%)	126 (15.1%)	366 (37.2%)	332 (75.3%)
Antihypertensive	96 (14.8%)	236 (28.2%)	533 (54.2%)	345 (78.2%)
Antiplatelet/anticoagulant	15 (2.3%)	69 (8.3%)	180 (18.3%)	279 (63.3%)

Data are number (percentage). QRISK2 is the 10-year future calculated cardiovascular risk.

The relationship between graded vascular risk and vascular disease, and the clinical characteristics of PD was analysed in the 2611 cases without any features that might possibly indicate an alternative diagnosis than PD (table 4). This meant that 298 cases were excluded from this analysis (10.2% of the 2909 study group). The analysis also excluded cases with missing results for disease duration (n=6, 0.2%), UPDRS 3 (n=189, 7.2%), motor subtype (n=185, 7.1%), MoCA (n=157, 6.0%) and LEDD (n=20, 0.8%). Additional analysis using multiple imputation of this missing data showed no qualitative differences, aside from the LEDD comparison in statin versus non-statin users (results not shown).

**Table 4:** Clinical correlates in 2611 recent-onset Parkinson's disease cases according to the presence of future cardiovascular risk and existing cardiovascular disease

Variable	QRISK2 <10% n=590 (22.6%)	QRISK2 ≥10% and <20% n=760 (29.1%)	QRISK2 ≥20% n=886 (33.9%)	Vascular disease n=375 (14.4%)	Unadjusted p value	Adjusted p value
Age	55.9 (7.0)	65.9 (4.5)	74.2 (5.3)	72.4 (7.6)	<0.001	<0.001*
Male sex	233 (39.5%)	482 (63.4%)	698 (78.8%)	284 (75.7%)	<0.001	<0.001†
Disease duration	1.3 (0.9)	1.3 (0.9)	1.3 (0.9)	1.4 (0.9)	0.05	0.78‡
UPDRS 3	20.3 (10.7)	22.2 (10.9)	25.9 (12.1)	26.8 (12.3)	<0.001	<0.001§
Motor subtype						
TD	299 (54.6%)	363 (51.0%)	397 (48.2%)	137 (40.1%)		
PIGD	177 (32.3%)	248 (34.8%)	330 (40.0%)	164 (48.0%)	<0.001	0.021§
Indeterminate	72 (13.1%)	101(14.2%)	97 (11.8%)	41 (12.0%)	0.60	0.069§
MoCA						
Total score	26.6 (2.7)	25.6 (3.2)	24.2 (3.6)	24.1 (3.5)	<0.001	<0.001§
Normal	486 (88.0%)	569 (79.7%)	511 (61.6%)	216 (60.3%)	<0.001	0.008§
MCI	38 (6.9%)	77 (10.8%)	147 (17.7%)	64 (17.9%)		
Dementia	28 (5.1%)	68 (9.5%)	172 (20.7%)	78 (21.8%)		
LEDD	273 (215)	287 (222)	291 (190)	314 (202)	0.004	0.069§
Smoking					<0.001	<0.001§
Non-smoker	413 (76.3%)	415 (58.9%)	411 (50.4%)	158 (47.7%)		
Ex-smoker	108 (20.0%)	265 (37.6%)	375 (46.0%)	160 (48.3%)		
Current smoker	20 (3.7%)	25 (3.5%)	30 (3.7%)	13 (3.9%)		

Data are mean (SD) or number (percentage) unless otherwise stated.

*Adjusted for disease duration and sex**.**

†Adjusted for age and disease duration**.**

‡Adjusted for age and sex**.**

§Adjusted for age, sex, disease duration and coffee intake.

LEDD, levodopa equivalent daily dose; MCI, mild cognitive impairment; MoCA, Montreal cognitive assessment; PIGD, postural instability gait difficulty; TD, tremor dominant; UPDRS 3, Movement Disorder Society unified Parkinson's disease rating scale part 3.

Increasing vascular risk was associated with increasing age (p<0.001) and an increasing proportion of men (p<0.001). Increasing vascular risk was also associated with worsening UPDRS 3 scores when adjusted for age, sex, disease duration and coffee intake (p<0.001), with UPDRS 3 scores ranging from 20.3 (SD 10.7) in those with a low QRISK2 score to 26.8 (SD 12.3) in those with established vascular disease. Increasing vascular risk was similarly associated with an increasing proportion with the PIGD phenotype (p=0.021) rising from 32.3% in those with a low QRISK2 score to 48.0% of cases with established vascular disease. Increasing vascular risk was also associated with increasing cognitive impairment; a worsening MoCA score (p<0.001) as well as an increasing proportion with MCI and dementia (p=0.008). The motor and cognitive characteristics of cases with high vascular risk were very similar to those seen in cases with established vascular disease (table 4). When we considered the potential effects of the interaction of sex on the clinical correlates of each vascular risk category, we did not find any statistically significant interactions (data not shown).

When we compared the clinical features of individuals treated versus untreated with statins (all indications), statin users had less PIGD (p=0.002) but a lower total MoCA score (p<0.001) and a greater proportion with cognitive impairment (p=0.010). Statin users also had a greater LEDD (p=0.035), but, as described earlier, this was not significant in our multiple imputation analysis (table 5).

**Table 5:** Clinical correlates in 2611 recent-onset Parkinson's disease cases according to the use of statin medication

Variable	Statin use, n=769	No statin use, n=1842	Unadjusted p value	Adjusted p value
Age	71.1 (7.4)	65.8 (9.4)	<0.001	0.16*
Sex	573 (74.5%)	1124 (61.0%)	<0.001	0.52†
Disease duration	1.3 (0.9)	1.3 (0.9)	0.34	0.96‡
UPDRS 3	25.3 (11.9)	23.0 (11.6)	<0.001	0.97§
Motor subtype				
TD	361 (50.1%)	835 (49.0%)		
PIGD	268 (37.2%)	651 (38.2%)	0.61	0.002§
Mixed	92 (12.8%)	219 (12.8%)	0.84	0.75§
MoCA				
Total	24.2 (3.7)	25.5 (3.3)	<0.001	<0.001§
Normal	450 (62.2%)	1332 (76.9%)	<0.001	0.010§
MCI	120 (16.6%)	206 (11.9%)		
Dementia	153 (21.2%)	193 (11.1%)		
LEDD	291 (209)	288 (207)	0.71	0.035§
Smoking				
Non-smoker	352 (50.7%)	1045 (61.5%)	<0.001	0.82§
Ex-smoker	319 (46.0%)	589 (34.7%)		
Current smoker	23 (3.3%)	65 (3.8%)		

Data are mean (SD) or number (percentage) unless otherwise stated.

*Adjusted for disease duration, sex and presence of existing cardiovascular disease or future cardiovascular risk.

†Adjusted for age, disease duration and presence of existing cardiovascular disease or future cardiovascular risk.

‡Adjusted for age, sex and presence of existing cardiovascular disease or future cardiovascular risk.

§Adjusted for age, sex, disease duration, coffee use and presence of existing cardiovascular disease or future cardiovascular risk.

MCI, mild cognitive impairment; MoCA, Montreal cognitive assessment; PIGD, postural instability gait difficulty; TD, tremor dominant; UPDRS3, Movement Disorder Society unified Parkinson's disease rating scale part 3.

When we compared the clinical features of those using and not using statins in individuals with established vascular disease and a QRISK2 score ≥10% (table 6), those treated with statins in the QRISK2 ≥10% subgroup had less PIGD (p=0.009), but also lower total MoCA scores and a greater proportion with cognitive impairment (p<0.001). Conversely in those with established CVD, those treated with statins were better cognitively, but with no significant differences in the proportion with PIGD.

**Table 6:** Clinical correlates in 2611 recent-onset Parkinson's disease cases according to the use of statin medication stratified by the presence of future cardiovascular risk and existing cardiovascular disease

	Vascular disease, n=375				QRISK2 ≥10%, n=1646			
Variable	Statin use, n=280	No statin use, n=95	Unadjusted p value	Adjusted p value	Statin use, n=450	No statin use, n=1196	Unadjusted p value	Adjusted p value
Age	72.5 (7.2)	72.0 (8.7)	0.57	0.60*	71.4 (6.6)	70.0 (6.4)	<0.001	<0.001*
Sex	221 (78.9%)	63 (66.3%)	0.014	0.017†	340 (75.6%)	840 (70.2%)	0.033	0.004†
Disease duration	1.4 (1.0)	1.2 (0.8)	0.071	0.078‡	1.3 (1.0)	1.3 (0.9)	0.76	0.60‡
UPDRS 3	26.8 (12.3)	26.5 (12.2)	0.82	0.89§	24.9 (11.6)	23.9 (11.7)	0.17	0.81§
Motor subtype								
TD	108 (42.0%)	29 (34.1%)			230 (54.1%)	530 (47.7%)		
PIGD	116 (45.1%)	48 (56.5%)	0.11	0.16§	144 (33.9%)	434 (39.1%)	0.031	0.009§
Mixed	33 (12.8%)	8 (9.4%)	0.82	0.58§	51 (12.0%)	147 (13.2%)	0.22	0.39§
MoCA								
Total	24.2 (3.6)	23.9 (3.3)	0.48	0.23§	24.0 (3.8)	25.1 (3.4)	<0.001	<0.001§
Normal	169 (63.3%)	47 (51.6%)	0.095	0.043§	249 (59.6%)	831 (73.8%)	<0.001	<0.001§
MCI	42 (15.7%)	22 (24.2%)			75 (17.9%)	149 (13.2%)		
Dementia	56 (21.0%)	22 (24.2%)			94 (22.5%)	146 (13.0%)		
LEDD	317 (210)	304 (176)	0.57	0.57§	275 (203)	294 (206)	0.11	0.025§
Smoking								
Non-smoker	109 (44.7%)	49 (56.3%)	0.12	0.20§	215 (52.1%)	611 (55.1%)	0.31	0.41§
Ex-smoker	127 (52.0%)	33 (37.9%)			183 (44.3%)	457 (41.2%)		
Current smoker	8 (3.3%)	5 (5.7%)			15 (3.6%)	40 (3.6%)		

Data are mean (SD) or number (percentage) unless otherwise stated.

*Adjusted for disease duration and sex.

†Adjusted for age and disease duration.

‡Adjusted for age and sex.

§Adjusted for age, sex, disease duration and coffee use.

MCI, mild cognitive impairment; MoCA, Montreal cognitive assessment; PIGD, postural instability gait difficulty; TD, tremor dominant; UPDRS 3, Movement Disorder Society unified Parkinson's disease rating scale part 3.

## Discussion

We have found, in a large study of recent-onset PD, that over 60% of cases have increased cardiovascular risk (without a history of vascular disease) that places them in a recommended treatment category for statin therapy. However, only around a quarter of those cases (27.0%) were prescribed such therapy. In contrast, fewer patients (around 15%) have manifest CVD, but a much larger proportion of these cases (75.3%) are prescribed statins. Given that statins are indicated in both groups,^11^ the comparatively lower usage of statins in those with increased vascular risk, compared with those with manifest CVD, suggests that the assessment and/or treatment approach to vascular risk is fundamentally different from that of manifest vascular disease, in recent-onset PD patients. The clinical relevance of this is suggested by the significant association of vascular risk, in addition to established CVD, with greater motor severity, including more axial features and gait problems, and with cognitive problems, including mild cognitive impairment and dementia.

The effects we have observed relating vascular risk to clinical phenotype in PD extend the evidence linking vascular risk factors with worse neurological status in prior smaller studies. PD cases with diabetes had worse global cognition,^5^ greater axial impairment^6^ and more rapid progression in terms of motor scores,^7^ while PD cases with hypertension had worse executive function and delayed memory.^4^ Further, carotid artery intima-medial thickness (a marker of subclinical vascular disease) correlated with higher levels of motor and cognitive impairment;^26^ and the presence of one or more vascular risk factor was associated with greater cognitive impairment and motor severity.^27^ Similar observations relate vascular risk to worse neurological status in Alzheimer's disease (AD)^28^ and multiple sclerosis,^29^ so these effects in PD are not unique.

Our use of a combined cardiovascular risk assessment tool is the subject of only one other PD study^30^ ^31^ to the best of our knowledge, which used a simplified version of the Framingham risk score. In that study, in 61 cases with an elevated Framingham risk, the Timed Up and Go test was significantly slower than in 22 cases with normal scores,^30^ and increased Framingham scores correlated with the axial motor impairment,^31^ again in keeping with the relationship between vascular risk and motor pattern in our study. Unlike the current study however, they found no relationship between vascular risk and motor or cognitive scoring, perhaps because of their longer PD duration (5.5 years) and considerably smaller study size.^30^ Considering dopaminergic therapy, one recent study found that PD cases with preceding diabetes were prescribed larger doses than cases without preceding diabetes.^32^ We did not find any association between LEDD and vascular risk, even after adjustment for patient age (which may influence drug dosage), despite their greater motor severity. We plan to test whether this is associated with lesser dopaminergic responsiveness in patients with vascular risk, in the ongoing follow-up phase of our study, which includes formal measurement of the L-dopa response.

The usage of statins in PD can be compared with that in other patient groups, although there are differences in methodology between the studies. In UK primary care, 80% of high vascular risk cases (Framingham score >20%) were treated with statins, which is more than double the 37.2% rate in our PD cases with similar risk (QRISK2 ≥20%). However, in that study, only 43% of individuals had a calculable vascular risk, and the 80% proportion on statins refers to this subset of cases and is therefore artificially high.^13^ In a Swiss study, lipid-lowering therapy was prescribed in 71% of high-risk cases, although this included a mix of cases with established CVD and high vascular risk scores.^18^ That study also described a medium-risk group (10–20% vascular risk), of whom 48% were prescribed statins, much higher than our rate of 15.1% for a comparable risk level. Although based on disease markers rather than calculated risk, higher statin usage was also reported in a European cross-sectional study: 42.2% with diabetes and 47.0% with high cholesterol.^16^

The rate of statin use in our PD cases with established CVD (75.3%) is very similar to the 74% rate of statin use for comparable cases in UK primary care,^13^ and higher than that observed in other settings: 68.6% of Irish community-living adults,^17^ and 43.4% of Italian patients after myocardial infarction.^15^

Overall, there appear to be higher rates of statin use in cases with manifest CVD than cases with increased vascular risk, which matches our findings in PD cases. Some more specific PD factors may influence this pattern. First, it is possible that the low cigarette smoking rate in PD cases may mislead the patient or clinician when considering vascular risk. Second, muscle cramps are a recognised barrier to statin use and occur in 5% of the general population,^33^ but may be contributed by PD symptoms; there may be a lower tolerance to statins in patients with such symptoms and increased vascular risk, compared with those with manifest CVD. Preliminary findings from the Parkinson's Pain Study suggest that around 13% of early PD patients experience painful cramps (Dr Monty Silverdale, personal communication) which may affect statin use. Although the complexity of many antiparkinsonian drug regimens, and other comorbidities, can lead to a high pill burden, which is known to affect medication usage, this does not readily explain the differences in statin use comparing our vascular risk and CVD cases. It may be that, in the absence of manifest CVD, clinicians primarily focus on PD rather than considering opportunistic vascular preventive treatments. Further analysis of reasons for the non-implementation or early cessation of statins in PD was beyond the scope of the current project, but merits specific study.

The potential implications of undertreating vascular risk in PD require consideration. If vascular risk is undertreated in PD as a result of statin underutilisation, this could influence the frequency of vascular disease seen in a PD population, which may explain some of the variability in studies comparing the frequency of CVD and risk factors in PD and controls. This could in turn influence conclusions relating to the aetiological role of vascular disease in PD. Vascular preventive therapy primarily reduces acute vascular events, for example, stroke and myocardial infarction,^11^ but may also limit chronic vascular damage. Following stroke, statins reduced white matter hyperintensity progression rates, and limited the decline in executive function.^34^ In AD, observational data suggested that treating vascular risk factors altered the rate of cognitive decline, although one can argue such studies are subject to bias.^35^ Improving the implementation of such treatments may therefore limit a vascular component of motor and cognitive deterioration in PD. However, there are limited data on such effects in PD. In one study, the presence of hypertension in PD patients correlated with greater Hoehn and Yahr stage progression over 5 years.^27^ In the ongoing follow-up phase of our study, we will test whether vascular risk and vascular disease contribute to the evolution of phenotype from TD to PIGD found in earlier longitudinal PD studies.^36^

The observation that individuals with the same vascular risk, but who were treated with statins, had a smaller proportion with the PIGD phenotype than those untreated with statins may lend support to the potential benefits of treating established CVD and elevated cardiovascular risk in PD. However, in those with a QRISK2 score ≥10%, those treated with statins had more cognitive impairment compared with those who were untreated. One possible explanation for this is a positive selection bias for starting statins, or maintaining them, when cognition is more impaired, or risks are perceived to be greater (such as in men). Although there are potential mechanisms whereby statins might increase cognitive impairment (through interference with myelin formation and function,^37^ and reduction in coenzyme-Q_10_ levels leading to impaired mitochondrial functioning and increased oxidative stress^38^), a recent systematic review and meta-analysis did not find any evidence of significant adverse effects of statins on cognition, either in cognitively normal participants or in those with AD.^39^ In addition, a reverse effect was seen in our study in those with established vascular disease, where those *not* using statins were cognitively worse. Given that our study is observational, we cannot reach definite conclusions and therefore the results of the Simvastatin as a Neuroprotective Treatment for Moderate Parkinson's Disease (PD STAT) randomised placebo-controlled trial will be of major importance (ClinicalTrials.gov identifier NCT02787590).

One limitation of our study is that the NICE guideline threshold was amended (from QRISK2 ≥20% to QRISK2 ≥10%) in July 2014,^11^ which overlapped with our study recruitment. For this reason, we stratified our results by the prior 20% threshold (published in 2008) and the newer 10% threshold. However, even considering the 20% threshold (37.2% prescribed statins), there was a major difference in statin use compared with that in manifest CVD (75.3% prescribed statins). A further limitation of the current study is that neuroimaging (eg, MRI or CT) was not used to assess vascular changes. While we have explored imaging in relation to individual risk factors elsewhere, our desired focus within this study was on the clinical assessment of vascular risk. We did not record statin dosage information, but this is in keeping with many studies which have explored statin use according to vascular risk indication,^13^ ^16^ ^17^ and with many studies examining statin use in relation to the risk of PD.^40^ A final limitation of our study relates to diagnostic accuracy, which like other early PD studies was based on clinical diagnosis in a research framework using clinical diagnostic criteria. Although some cases may evolve to an alternative diagnosis, in most cases, vascular risk assessment and appropriately directed treatment remain relevant. For this reason, we included all patients in our assessment of vascular risk and treatment, and limited our analysis to patients without possible atypical features only when examining clinical correlates between vascular risk and disease, and PD phenotype.

In conclusion, a large proportion of individuals with recent-onset PD have increased cardiovascular risk, which is associated with greater motor and cognitive severity, and greater axial impairment. Statin therapy is underused in these PD cases, which contrasts with much high rates of statin use in PD cases with manifest CVD. Increasing the usage of statins in PD patients with increased vascular risk would reduce acute cardiovascular events, but might also reduce chronic vascular damage, and thereby slow the progression of motor and cognitive decline. Patients with PD have regular visits to healthcare providers, and so greater awareness and increased intervention in this group could have an immediate impact in a large population.
